# Investigation on acquired palbociclib resistance by LC-MS based multi-omics analysis

**DOI:** 10.3389/fmolb.2023.1116398

**Published:** 2023-01-19

**Authors:** Zhichao Xue, Jiaming Zeng, Xinchi Yin, Yongshu Li, Bo Meng, Yang Zhao, Xiang Fang, Xiaoyun Gong, Xinhua Dai

**Affiliations:** ^1^ Technology Innovation Center of Mass Spectrometry for State Market Regulation, Center for Advanced Measurement Science, National Institute of Metrology, Beijing, China; ^2^ College of Chemical Engineering, Shenyang University of Chemical Technology, Shenyang, China; ^3^ Shenzhen Institute for Technology Innovation, National Institute of Metrology Shenzhen, Shenzhen, China

**Keywords:** LC-MS, proteomics, Glyproteomics, drug resistance, cancer biology

## Abstract

Palbociclib is a specific CDK4/6 inhibitor that has been widely applied in multiple types of tumors. Different from cytotoxic drugs, the anticancer mechanism of palbociclib mainly depends on cell cycle inhibition. Therefore, the resistance mechanism is different. For clinical cancer patients, drug resistance is inevitable for almost all cancer therapies including palbociclib. We have trained palbociclib resistant cells *in vitro* to simulate the clinical situation and applied LC-MS multi-omics analysis methods including proteomic, metabolomic, and glycoproteomic techniques, to deeply understand the underly mechanism behind the resistance. As a result of proteomic analysis, the resistant cells were found to rely on altered metabolic pathways to keep proliferation. Metabolic processes related to carbohydrates, lipids, DNA, cellular proteins, glucose, and amino acids were observed to be upregulated. Most dramatically, the protein expressions of COX-1 and NDUFB8 have been detected to be significantly overexpressed by proteomic analysis. When a COX-1 inhibitor was hired to combine with palbociclib, a synergistic effect could be obtained, suggesting the altered COX-1 involved metabolic pathway is an important reason for the acquired palbociclib resistance. The KEGG pathway of N-glycan biosynthesis was identified through metabolomics analysis. N-glycoproteomic analysis was therefore included and the global glycosylation was found to be elevated in the palbociclib-resistant cells. Moreover, integration analysis of glycoproteomic data allowed us to detect a lot more proteins that have been glycosylated with low abundances, these proteins were considered to be overwhelmed by those highly abundant proteins during regular proteomic LC-MS detection. These low-abundant proteins are mainly involved in the cellular biology processes of cell migration, the regulation of chemotaxis, as well as the glycoprotein metabolic process which offered us great more details on the roles played by N-glycosylation in drug resistance. Our result also verified that N-glycosylation inhibitors could enhance the cell growth inhibition of palbociclib in resistant cells. The high efficiency of the integrated multi-omics analysis workflow in discovering drug resistance mechanisms paves a new way for drug development. With a clear understanding of the resistance mechanism, new drug targets and drug combinations could be designed to resensitize the resistant tumors.

## 1 Introduction

Targeted drugs have fewer side effects and higher potency than traditional chemodrugs (Zhong et al., 2021). However, since the targeted drug relied on one or several specific target proteins or pathways, it is inevitable to develop drug resistance (Liu W. J. et al., 2020; Pancholi et al., 2020; Kanbayashi et al., 2021). Due to the heterogeneity of tumors and the individual difference between patients, the mechanisms behind drug resistance are different. It is of great importance to establish fast and high throughput methods to identify and verify the resistance mechanism for each specific drug resistance case. Specific CKD4/6 inhibitor palbociclib can target the proliferation of cancer cells by cell cycle inhibition, it is a targeted drug with fewer side effects and high efficiency which was approved for metastatic breast cancer at first (Finn et al., 2016), and it is widely used for clinical and preclinical studies in multiple tumor types including, hepatocellular carcinoma (Bollard et al., 2017), ovarian cancer (Konecny et al., 2011), rhabdoid tumor (Katsumi et al., 2011), glioblastoma (Cen et al., 2012) nasopharyngeal carcinoma (Xue et al., 2020), as well as colorectal cancer (Zhang et al., 2017). After FDA approval, palbociclib has been applied to patients for around 15 years, and numerous drug-resistance cases have been reported (Turner et al., 2018; Pandey et al., 2019; Montaudon et al., 2020; Pancholi et al., 2020).

Attempts have been made to discover the potential reason for palbociclib resistance, there are three major kinds of studies. The first kind is retrospective analysis. Through mRNA expression analysis or IHC stains of patient samples, researchers found that overexpress of cyclin E was associated with palbociclib resistance (Turner et al., 2019). With patient samples, the finding from this kind of study was considered to be the most reliable. However, the limitation was also obvious, as the clinical samples were very precious, not easy to be obtained by clinical doctors no mention for biomedicine researchers. The second kind was a genomics and transcriptomics study with preclinical models, researchers found that the resistance could be contributing to the up-regulation of the cyclin E-CDK2 pathway and several immune-related genes (Pandey et al., 2021). However, there were also two major limitations of this kind of study. They did not conduct *in vitro* or *in vivo* studies to verify their findings. Besides, due to the complex post-translational modifications, the up or downregulated genes or mRNA screened out at the genomic level may not finally turn out to be functional proteins. The last kind includes *in vitro* studies carried out by generating palbociclib resistance cells. In this kind, traditional techniques such as western blot and QPCR were mostly used to verify well-known targets. Such as in Fu’s and Herreras’ studies (Herrera-Abreu et al., 2016; Fu et al., 2022), their target ABCB1 and Cyclin E were selected based on experience and references. They mainly focused on testing and verifying known mechanisms, hardly identifying *de-novo* mechanisms to offer new solutions to drug resistance.

Label-free LC-MS based multi-omics study could integrate the quantitative data of biological functional cellular molecules and their modification information in a high throughput way. Proteomic, metabolomic as well as glycoproteomic studies could offer detailed whole-cell protein, metabolite, and glycoprotein data quantitatively for the complex mechanism analysis (Pan et al., 2021; Zeng et al., 2021; Zhao et al., 2021). Firstly, proteins constitute the vast majority of drug targets, the use of proteomics to study the interrelationships of protein expressions in drug treatments can provide important insights into drug development and facilitate drug clinical use (Li et al., 2020; Li and Zhu, 2020; Mateus et al., 2022). However, protein changes are not the only clue for cells that develop drug resistance, accumulating evidence suggests that cancer metabolism is intimately linked to drug resistance (Rahman and Hasan, 2015), and determining the metabolic profile of a drug is a critical part for investigate drug resistance (Li et al., 2012). Besides, the post-transcription of proteins also matters, as one of the most common post-transcriptome modifications in proteins, glycosylation accounts for more than 50% of known eukaryotic proteins. N-glycosylation plays an important role in understanding various cancer mechanisms and it provides a set of targets for diagnostic applications and therapeutic strategies (Taniguchi et al., 2009; Silsirivanit, 2019; Haga and Ueda, 2022). High-throughput proteomics, metabolomics as well as glycoproteomics based on the LC-MS technique could provide us with a way to evaluate thousands of important interrelated proteins, metabolites, and glycoproteins with high efficiency (Waniwan et al., 2018). Besides modifications of protein, the modifications of DNA and RNA could also be investigated by LC-MS based methods (Fang et al., 2022; Hu et al., 2022). The integration analysis of LC-MS based multi-omics was a powerful tool for interpreting drug mechanisms, including drug resistance, drug efflux, and drug sensitivity (Cuperlovic-Culf and Culf, 2016; Clarke et al., 2017; Ji et al., 2017; Waniwan et al., 2018; Zhang et al., 2019). Furthermore, with the establishment of free-access online platforms for bioinformatic analysis, such as the WuKong platform and MetaboAnalyst (Pang et al., 2022; Yang et al., 2022), biomedicine researchers and clinical doctors without professional bioinformatics skills can also perform bioinformatics analysis easily.

In this study, we trained a colorectal cancer cell line HCT116 to acquire palbociclib resistance, then we use LC-MS based multi-omics studies and open-platform bioinformatic analysis to disclose the mechanism behind this resistance. One purpose of this study is to identify new targets for acquired palbociclib resistance. More importantly, we set this investigation as an example to establish an LC-MS based approach to discover and verify drug resistance with high efficiency. The LC-MS based proteomics, metabolomics, and glycoproteomics as independent techniques could detect a lot of changes behind cellular signaling, disease development, and resistance acquisition separately. In this study, new findings with biological verification have been obtained when we combined these three techniques. A significant upregulation of COX-1 at the protein level, an alteration of the metabolic pathway involving N-glycan biosynthesis, as well as a global upregulation of N-glycosylation in resistant cells were observed, all these findings suggested that the development of drug resistance happening within the cells was multi-dimensional, the changes in protein level, metabolites level, and glycosylation modification level were complex and interrelated. Deciphering the complicated interactions between these changes could now be realized and offer us the chance to understand more about drug resistance. Here we suggested that the high efficiency in LC-MS omics analysis could enhance the new mechanism exploration behind drug resistance with high-efficiency identification and simplicity of operation, which could be applied to individual patients’ drug-resistant cases thus enhancing the precise medicine.

## 2 Materials and methods

### 2.1 Cell culture and reagents

HCT116 cells obtained from BNCC (#287750) were cultured at a 37°C incubator with a 5% CO_2_ supply. The culture medium consisted of 90% RPMI-I640 (Solarblo, #31800) and 10% FBS (Every Green, #11011-8611). Palbociclib (Selleck Chemicals, #S1116) was dissolved in distilling H_2_O at the stock concentration of 10 mM and was further diluted to a working concentration with the cell culture medium. Cisplatin (Sigma-Aldrich, RAB7778) was dissolved with distilling H_2_O to a stock concentration of 6.67 mM and was further diluted with the cell culture medium to the working concentration. SC-560 (Selleck Chemicals, #S6686) was dissolved in DMSO to a stock concentration of 10 mM. Tunicamycin (Aladdin, #T101151) was dissolved in DMSO to a stock concentration of 1 mM. Kifunensine (Aladdin, #K274698) was dissolved in H_2_O to a stock concentration of 1 mM. HCT116 palbociclib resistance cell (HCT116 PD_R or resistant cells) was established by slowly increasing palbociclib in the culture medium for over 6 months.

### 2.2 CCK8 assay

3000 cells of HCT116/HCT116 PD_R were seeded in 96-well plates overnight before drug treatment. CCK8 (Cell Counting Kit-8, Biorigin, #BN15201) assay was hired for cell viability measurement at the end of drug treatments. CCK8 solution was added into the wells 1:10 v/v and then the plates were incubated for 1.5 h at 37°C, the optical density was measured at 490 nm and the OD490 value was the corresponding viability value. Growth inhibition in each well was calculated as (viability_control_ - viability_drug_)/viability_control_ × 100%.

### 2.3 Proteomic study

For the proteomics study, HCT116/HCT116 PD_R cells were seeded in 100 mm dishes for four repeats. Cells were collected for the sample preparation step. Proteins were extracted from the cells by Ripa buffer (Solarblo, #R0020-100). For each sample, 500 μg proteins were added into a 30 KD ultrafiltration tube for filter-aided sample preparation (FASP) digestion. After washing the proteins with 8 M UA (Urea Sigma-Aldrich, #SLCB4221), the reduction reaction was conducted by adding 100 μL of 20 mM DTT (1,4-Dithiothreitol, SIGMA, #SLCF2685) to the proteins and incubated at 37°C for 4 h, and then centrifuged for 15 min at 14,000 r/min to discard the solution. 100 μL of 50 mM IAA (3-Indole acetic acid, SIGMA, #SLCC6164) was added for alkylation afterward. The mixture was incubated for 30 min at room temperature in the dark and centrifuged for 15 min at 14,000 r/min to discard the solution. Afterward, 200 μL of 8 M UA and NH_4_HCO_3_ (Sigma-Aldrich, #BCBV0122) were successfully added three times for washing. Finally, trypsin (Enzyme and Spectrum, #P01001) (Enzyme mass: Protein mass = 1: 25) was added, and the proteins were incubated at 37°C for 16 h, peptide samples were collected by centrifuge at 14,000 r/min for 15 min, and then 100 μL of ddH_2_O and 200 μL of NH_4_HCO_3_ were added to elute the samples respectively, and centrifuged at 45 °C for hot drying, waiting for loading.

DDA (Data Dependent Acquisition) mode was used to collect data from the samples, and each sample was separated and detected by Easy-nLC 1,200 tandem Orbitrap Fusion Lumos (Meyer, 2019). After dilution of the sample with 0.1% formic acid, the concentration of peptides was measured by ultramicro spectrophotometer, and the loading volume was 1 μg. The inner diameter of the separation column was 75 μm, the length was 25 cm (reprosil-pur c18-AQ, 1.9 μm; Dr. Maisch), mobile phase: phase A was 0.1 %FA-H_2_O; Phase B is 0.1% FA-80 %ACN; elution gradients: 0–5 min (4%–10% B), 5–48 min (10%–22% B), 48–66 min (22%–35% B), 66–76 min (35%–90% B), 76–78 min (90% B). MS acquisition conditions were set as follows: fragmentation mode was high-energy collision dissociation (HCD), ionization mode was nano-ESI, and positive ion mode was used for scanning. For primary mass spectrometry, the ion scan range is 350–1,550 m/z, the resolution is 120,000, the Automated gain control (AGC) is 40,000, and the maximum ion injection time is 50 ms. For the secondary scan, the resolution was 15,000, the normalized collision energy was 30%, the AGC was 50,000, the maximum ion implantation time was 50 ms, and the mass data were acquired in real-time by XCalibur.

For data analysis, the tandem mass spectra were searched against the human UniProt database (version 20210902, 20,375 sequences) using MaxQuant (version 1.6.12.0). Trypsin was selected as the proteolytic enzyme, and two missed cleavage sites were allowed. Cysteine carbamidomethylation was set as the fixed modification. The oxidation of M and acetylation of the protein N-terminal was set as the variable modifications. The first search mass tolerance was 20 ppm, and the main search peptide tolerance was 4.5 ppm. The false discovery rates of the peptide–spectrum matches (PSMs) and proteins were set to less than 1%.

### 2.4 Metabolomic study

For metabolomics analysis, HCT116/HCT116 PD_R cells were seeded in 6-well plates with 80% confluence were collected. For sample preparation, the cells were first rinsed with PBS. Then 1.2 mL of 50% MeOH was added into each well for extraction of intracellular metabolites. Samples were completely lysed using an ultrasonic homogenizer in an ice bath for 30 min and centrifuged at 14,000 rpm under 4°C for 5 min.

The supernatants were analyzed by liquid chromatography-mass spectrometry (LC-MS) simultaneously after centrifugation. 100 μL of the supernatant was transferred to a new vial and analyzed by a Thermo U3000-Orbitrap Elite liquid chromatography-mass spectrometer (LC-MS) system equipped with an electrospray ionization (ESI) source. liquid chromatography was performed on a Waters XBridge BEH Amide column (150 × 2.1 mm, 2.5 µm particle size, Waters Corporation, Milford, MA). The mobile phase for chromatographic separation was composed of phase A was 10 mM ammonium hydroxide, 10 mM ammonium acetate in 95% ACN/5% H_2_O; and phase B was 10 mM ammonium hydroxide, 10 mM ammonium acetate in 95% H_2_O/5% ACN. Elution gradients: 0–1 min (90% B), 1–11 min (90%–40% B), 11–15 min (40% B), 15–17 min (40%–90% B). The ionization mode was ESI, and the positive ion mode was used for scanning. 10 μL of each sample was injected for positive ion electrospray ionization analysis. The data analysis was conducted by MetaboAnalyst 5.0, the raw MS files were uploaded and analyzed automatically (Pang et al., 2022).

For direct mass spectrometry detection of ATP and L-glutathione, nano-ESI in negative polarity was applied as described in Tan’s study (Tan et al., 2022).

### 2.5 Glycoproteomic study

For glycoproteomic analysis, HILIC material was used to enrich glycopeptides and Venusil HILIC (5 μm, 100 Å) was activated by washing 3 times with 0.1% TFA and 80% acetonitrile/0.2% TFA for 10 min. After adding 100 μL of 80% acetonitrile/0.2% TFA solution, transfer the activated packing to the diluted peptide and then spin the reaction for 2 h at room temperature. Load the suspended solids onto a pipette tip fitted with a C8 membrane (Axygen, Inc., Union City, California, United States) and wash twice with 80% acetonitrile/0.2% TFA. Collect intact N-glycopeptides by eluting three times with 0.1% TFA eluent, combine the eluate, then dry with a SpeedVac centrifuge at 45°C (Eppendorf, concentrator plus) and store at −80°C for further LC-MS/MS analysis.

For LC-MS/MS detection, the mobile phase for chromatographic separation was set for proteomics. 0.5 μg of N-glycopeptides reconstituted in 0.1% FA were separated over a gradient of 78 min at a flow rate of 300 L/min (0–8 min, 5–8% B; 8–58 min, 8–22% B; 58–70 min, 22–32% B; 70–71 min, 32–90% B; 71–78 min, 90% B). For a full MS scan, the Orbitrap resolution was set to 120,000, with an AGC target value of 4 × 10^5^ for a scan range of 800–2,000 m/z and a maximum injection time of 100 ms. For MS_2_ scan, the HCD fragmentation was performed at the isolation width of 2 m/z and a segmented HCD collision energy of 20%, 30%, and 40%.

For glycoproteomic data analysis, the raw MS files of enriched N-glycopeptides were searched against the human Swiss-Prot database (version 20210902, 20,375 sequences) with pGlyco v. 2.2.253, as previously described by (Zhang. Y et al., 2020). The following parameters were used. Mass tolerances for the precursors and fragment ions were set as ±5 and ±20 ppm, respectively. Two missed cleavage sites were allowed for trypsin digestion. The fixed modification was carbamidomethylation of all cysteine residues (+57.02 Da). Variable modifications included the oxidation of methionine (+15.99 Da), deamidation of asparagine (+0.98 Da), and acetylation of the protein N-terminal (+42.01 Da). The N-glycosylation sequon (N-X-S/T/C; X ≠ P) was modified by changing “N” to “J”. Both of these had the same mass. Quality control methods for intact glycopeptide identification were set to the 1% glycopeptide–spectrum matches (GPSM) false discovery rate (FDR). Quantification information (MS1 peak intensity) of N-glycopeptides spectra was acquired from MaxQuant (Max Planck Gesellschaft, Munich, Germany) based on their unique MS/MS scan numbers from pGlyco 2.0 results.

### 2.6 Bioinformatics analysis

Bioinformatics analysis was performed by using “Wu Kong” which is an R language-based in-house freely available platform (https://wkomics.omicsolution.com/wkomics/main/). For quantitative analysis of the proteomics and glycoproteomics data, the LFQ intensities were extracted from the MaxQuant result file to represent the expression level of corresponding proteins in each sample. The intensities were normalized to the median for further statistical analysis. Limma analysis was applied to the normalized data to obtain differential expressed proteins (DEPs), and the resulting *p*-value and Fold Change value of proteins were then extracted for volcano plots. The significant threshold of volcano plots was set as curved with >0.585 and < −0.585, and the confidential interval was set to 0.95. The significant DEPs extracted from volcano plots were subjected to further Gene Ontology (GO) enrichment analysis and Kyoto Encyclopedia of Genes and Genomes (KEGG) pathway enrichment analysis using the “Wu Kong” platform. For metabolomics data analysis, the normalization, missing value imputation, and volcano plot (Fold Change >2, *p*-value <0.05) were all conducted within MetaboAnalyst 5.0. The KEGG enrichment of metabolomics was conducted through WuKong.

### 2.7 Transwell migration assays

Cell migration assay was conducted using a 24-well Transwell chamber with a polycarbonate membrane filter of 8 μm pore size (Falcon, Lot #353097). HCT116/HCT116 PD_R cells (5 × 10^5^) suspended in 100 μL serum-free RPMI-1640 media were seeded in the upper chamber, and 500 μL RPMI-1640 with 10% FBS was added to the lower well. Cells were incubated at 37°C with 5% CO_2_ for 24 h to allow migration, and the non-migrating cells in the upper chamber were carefully removed using a cotton swab. The migrated cells were then fixed with 100% methanol for 30 min at cold and stained with 4% crystal violet for 20 min at room temperature. After three times washing with tap water, the migrated cells on the lower surface of the chamber were imaged and photographed with an inverted microscope (OLYMPUS CKX53) at ×100 magnification in five randomly selected visual fields, and the migrated cells were counted using ImageJ software. Each assay was performed in four repeats, and the number of migrated cells was shown as means ± SD.

## 3 Results

### 3.1 Quantitative proteomic analysis of palbociclib resistant cells

**FIGURE 1:**
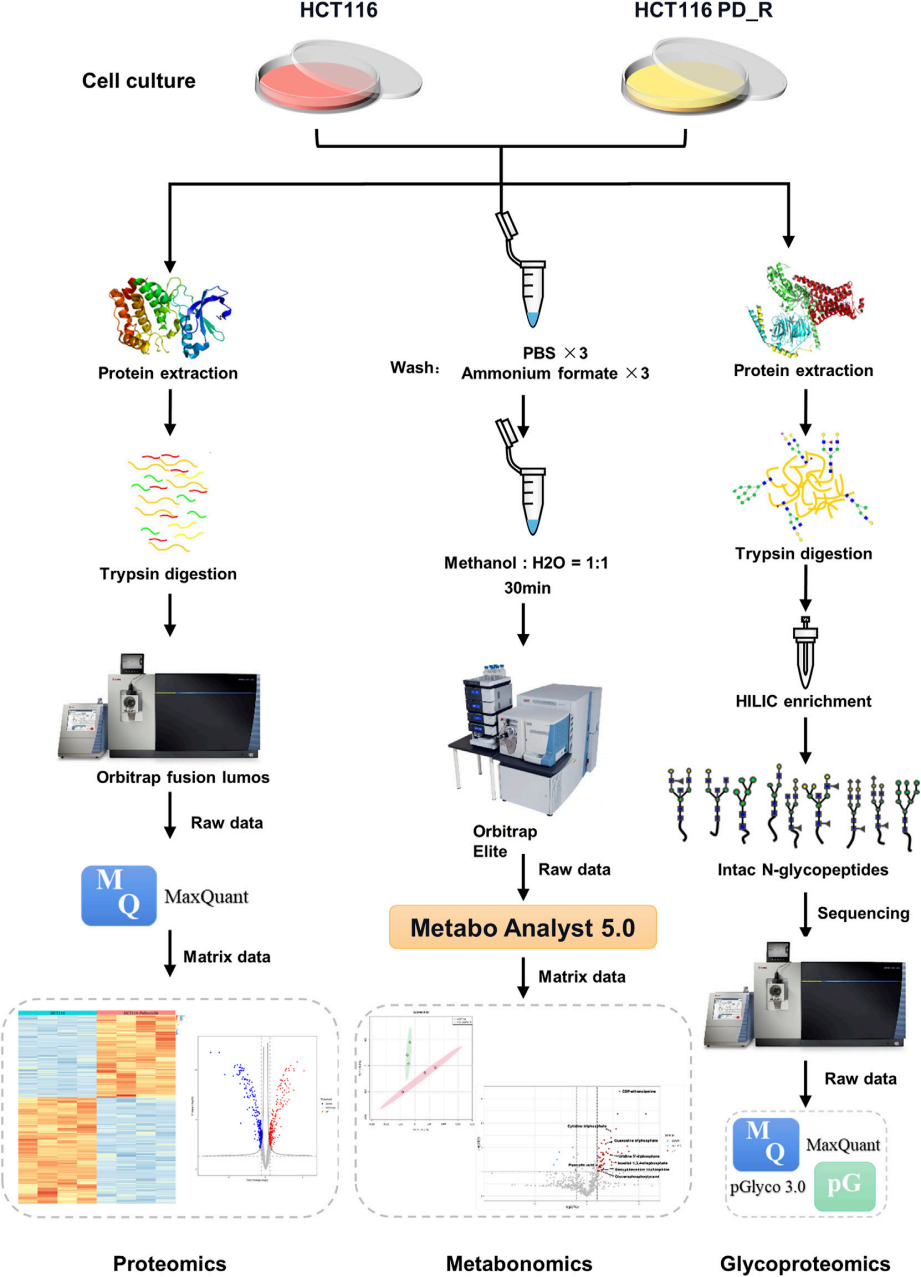
Workflow of LC-MS proteomics, metabolomics, and N-glycoproteomics analysis. HCT116 and HCT116 PD_R cells were collected and subjected to proteomics (left panel), metabolomics (middle panel), and N-glycoproteomics (right panel) analysis.

**FIGURE 2:**
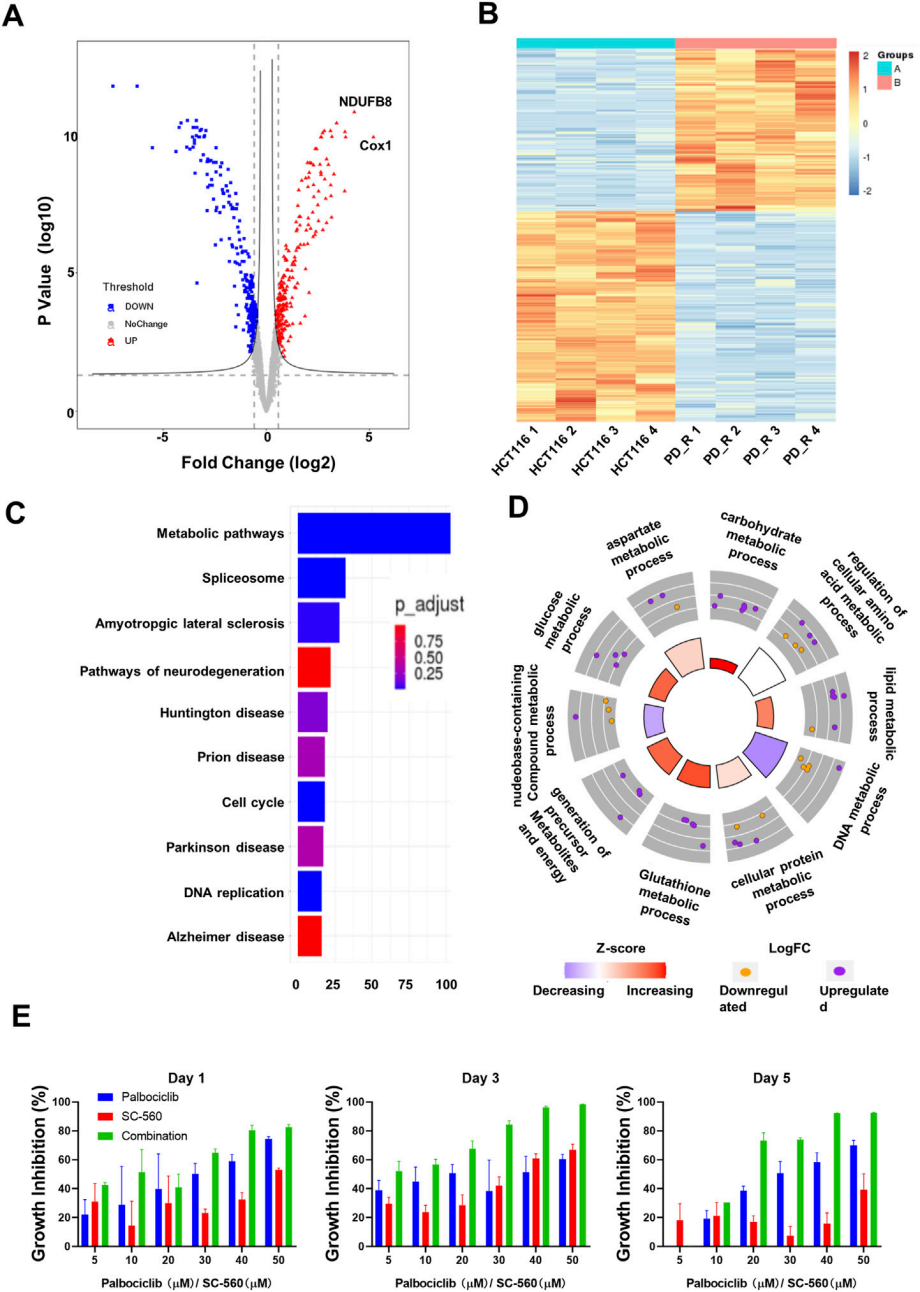
Quantitative proteomic analysis of HCT116 and HCT116 PD_R cells. **(A)** Volcano plot of proteomic data constructed using fold changes (HCT116 PD_R/HCT116) and adjusted *p* values. Red dots: significantly upregulated proteins. Blue dots: significantly downregulated proteins. Gray dots: proteins with no significant changes. **(B)** Heat map generated from the normalized intensity of the 526 DEPs across two study groups. **(C)** Top ten enriched KEGG pathways of the DEPs. **(D)** GO Circle plot of the top ten metabolic-related enriched biological processes of the DEPs between HCT116 PD_R *versus* HCT116. The upregulated (purple dots) and downregulated (blue dots) proteins in each process are distributed in the outer circle of the plot. The inner-circle displays the Z-score, calculated as the number of upregulated proteins minus the number of downregulated proteins divided by the square root of the total count. The larger Z-score represents more upregulated proteins enriched in the process. The color of the Z-score represents the increasing or decreasing expression of the process. **(E)** Growth inhibition rate of the combination treatment of SC-560 with palbociclib in HCT116 PD_R cells. Three-time points were designed as drug treatment for 1 day, 3 days, and 5 days respectively. Five doses of SC-560 (10, 20, 30,40, and 50 μM) were combined with five doses of palbociclib (10, 20, 30,40, and 50 μM). Data were represented as mean ± SD.

HCT116 palbociclib-resistant cells (HCT116 PD_R or resistant cells) were established by slowly increasing palbociclib in the culture medium from 0.1 μM to the final 10 μM for over 6 months. As a result, the IC50 of HCT116 PD_R has increased 60 times than the original cells Supplementary Figure S1. The drug resistance was not developed exclusively for palbociclib, the resistant cells also demonstrated 3 times resistance to cisplatin. To deeply discover the potential underlying mechanisms behind palbociclib resistance, the LC-MS/MS proteomic analysis was conducted at first to offer us whole-cell protein information (Figure 1). 4,344 proteins in total were identified in two study groups. Differently expressed proteins (DEPs) between the parental and resistant cells were compared through Limma analysis and presented as volcano plots in Figure 2A. The DEPs could be classified into two distinct clusters between parental and resistant cells, and the normalized LFQ intensities of the same protein were similar in the four biological replicates Figure 2B. There were 223 upregulated and 303 downregulated proteins identified in the resistant cells. The top 10 upregulated DEPs with the most significant Fold Changes were listed in Table 1. Notably, COX-1 and NDUFB8 were listed as the top two upregulated proteins, and they were both involved in oxidative phosphorylation (Supplementary Figure S2). COX-1 inhibitor SC-560 was tested in palbociclib-resistant and parental cells (Supplementary Figure S3), and the IC50 was observed to be significantly lower in the resistant cells. We further used SC-560 to combine with palbociclib in HCT116 PD_R as in Figure 2E, five doses of drug combination regimes and three-time points’ tests were designed. The result indicated that COX-1 inhibition could enhance the cell inhibition effect of palbociclib in resistant cells, suggesting a synergistic effect.

**TABLE 1:** Top ten upregulated DEPs from proteomics analysis.

Upregultaed Proteins					
Name	Fold.Change	P.Value	adj.P.Val	Protein names	Gene names
P00395	5.189497965	1.59E-12	1.25E-10	Cytochrome c oxidase subunit 1	COX-1
O95169	4.263333988	2.04E-14	1.55E-11	NADH dehydrogenase [ubiquinone] 1 beta subcomplex subunit 8, mitochondrial	NDUFB8
Q59GN2	3.844253496	1.43E-12	1.25E-10	Putative 60S ribosomal protein L39-like 5; 60S ribosomal protein L39	RPL39P5
O95810	3.809123133	6.02E-13	8.04E-11	Serum deprivation-response protein	SDPR
Q9NPL8	3.79772362	4.06E-10	1.11E-08	Complex I assembly factor TIMMDC1, mitochondrial	TIMMDC1
Q9NUQ2	3.40744242	1.19E-13	3.87E-11	1-acyl-sn-glycerol-3-phosphate acyltransferase epsilon	AGPAT5
P15104	3.274058438	7.42E-11	2.72E-09	Glutamine synthetase	GLUL
O75880	3.180485833	1.11E-12	1.20E-10	Protein SCO1 homolog, mitochondrial	SCO1
Q96I59	3.163047452	4.79E-09	9.90E-08	Probable asparagine--tRNA ligase, mitochondrial	NARS2
Q8NCA5	3.136577654	6.53E-10	1.67E-08	Protein FAM98A	FAM98A

To understand the functions these DEPs played in the development of drug resistance, KEGG pathway enrichment analysis and GO enrichment analysis were performed. There were more than 100 DEPs involved in metabolic pathways as in Figure 2C. Therefore, we analyze those metabolic-related pathways enriched in the GO biology process (BP) as in Figure 2D. GO circle plots of the top 10 enriched metabolism-related BPs were generated based on Fold Changes and the adjusted *p*-value of each involvement protein (Supplementary Table S4). The major enriched BPs and the composition of upregulated and downregulated proteins in each BP could be present clearly. As shown in the figure, the resistant cells had upregulated expression in metabolic pathways involving carbohydrates, lipids, glucose, glutathione, cellular protein, and aspartate.

### 3.2 Alternative metabolic pathways in resistant cells

**FIGURE 3:**
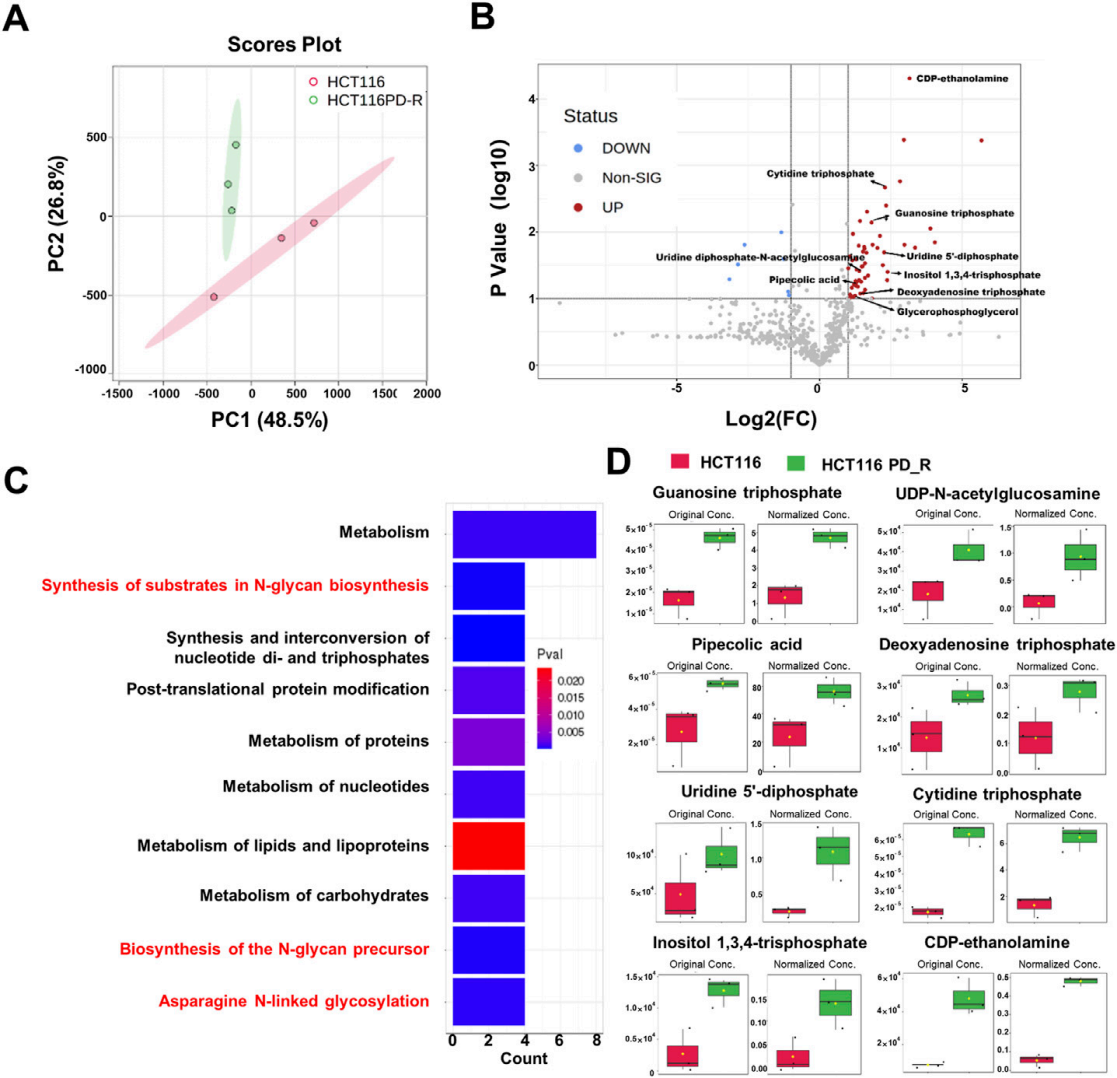
Metabolomics analysis of HCT116 and HCT116 PD_R cells. **(A)** Score plots of PCA models for the metabolome data obtained by LC-MS, showing the metabolic profile differences between HCT116 and HCT116 PD_R. Red cycle: HCT116 PD_R; Green cycle: HCT116. **(B)** Volcano plot of metabolomic data constructed using Fold Changes (HCT116 PD_R/HCT116) and adjusted *p*-values (FC > 2, *p*-value < 0.05). **(C)** Top ten enriched KEGG pathways of the upregulated metabolites. **(D)** Significantly upregulated metabolites among the HCT116 and HCT116 PD_R cells. The bar plots on the left show the original peak intensity values (mean ± SD). The bars on the right present the normalized values.

To deeply investigate the metabolism changes in palbociclib-resistant cells, metabolomics analyses were therefore applied. A total of 554 features were identified, based on the LC-MS data the PCA score plot showed an obvious separation between the two study groups (Figure 3A). No outlier detection was identified from the data overview. As indicated by the volcano plot (Figure 3B; Supplementary Table S5), the upregulated features induced by resistant cells were presented on the right-hand side of the valley, while the left-hand side of the valley represents those that were downregulated. There were 54 upregulated and seven downregulated features identified with a Fold Change >2. Within these different expression features, eight metabolites were annotated according to MetaboAnalyst 5.0 platform and artificially search against HMDB and MassBank. After enrichment of the upregulated metabolites against Reactome pathways (Figure 3C), we found that N-glycan biosynthesis was listed among the top 10 terms. Four metabolites including cytidine 5′-triphosphate (C00063), UDP-N-acetylglucosamine (C00043), uridine 5′-diphosphate (C00015) and guanosine 5′-triphosphate (C00044) were involved in N-glycan biosynthesis. The original concentration and normalized concentrations of these metabolites were presented in Figure 3D.

### 3.3 Global elevated N-glycosylation in resistant cells

**FIGURE 4:**
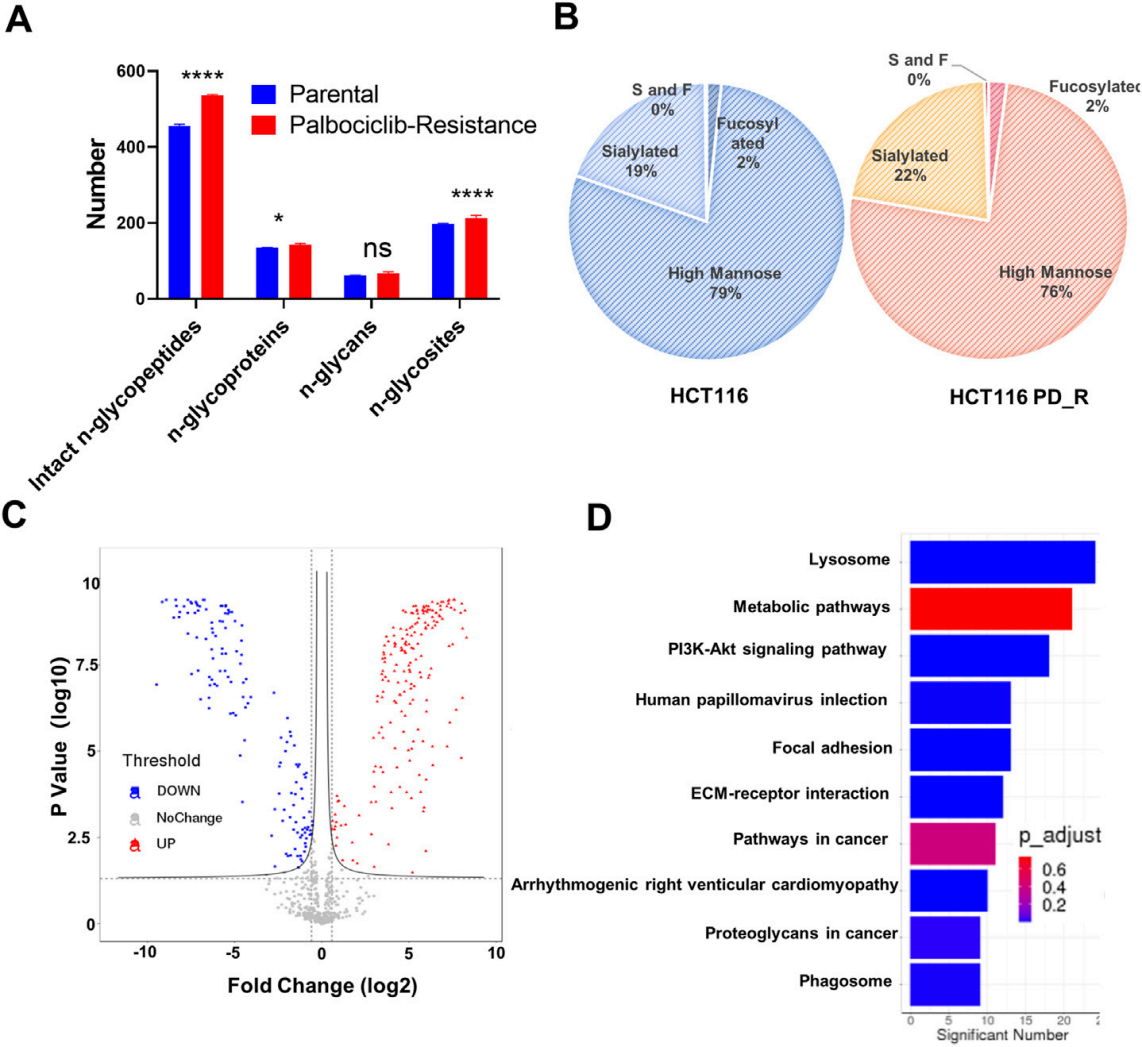
N-glycoproteomic analysis of HCT116 and HCT116 PD_R. **(A)** The number of identified N-glycoproteins, intact N-glycopeptides, N-glycosites, and N-glycans in two study groups. **(B)** The proportion of N-glycans of each type in two study groups. **(C)** Volcano plot of N-glycoproteomics data constructed using Fold Changes and *p*-value of IGPs. **(D)** Top ten enriched KEGG pathways of IGPs. (Statistical analysis was performed by Graphpad Prism 8.0, and the Two-Way ANOVA multiple comparisons were applied within each row. The significance of the differences was presented by starts: **** means *p*-value <0.0001, *** means *p*-value <0.001, ** means *p*-value <0.01, * means *p*-value <0.05, ns means no significant difference).

Asparagine-linked-glycans (N-glycans) are one of the most common and important post-translational modifications of proteins (Cao et al., 2018). As upregulated N-glycan biosynthesis and elevated Asparagine N-linked glycosylation (Figure 3C) were observed from metabolomic analysis, we further conducted N-glycoproteomic analysis to verify if N-glycosylation was involved in the development of palbociclib resistance. 7,395 intact-glycopeptides (IGPs) and 490 N-glycoproteins were identified and the numbers of intact-glycopeptides, N-glycan types as well as the N-glycosites for two study groups were compared in Figure 4A. As expected, the global N-glycosylation was higher in resistant cells than in parental cells. The average numbers of identified intact N-glycopeptides, N-glycosites and N-glycans were significantly higher in resistant cells. We also explored how the glycan compositions were distributed in two study groups. Regardless of their abundances, it was found that the proportion of sialylated N-glycan was higher in the resistant cells (22%) than in the parental cells (19%), sialylated N-glycan was a type of glycan modification closely related to the malignancy of the tumor (Figure 4B). We also compared the differentially expression glycopeptides (DEGs) between resistant and parental cells, there were 196 upregulated and 143 downregulated DEGs identified through the volcano plot (Figure 4C). It is noticeable that among the top 10 upregulated DEGs, four of them were sialylated type Table 2. Subjected to KEGG pathway enrichment, the top 10 terms involved by these DEGs were listed in Figure 4D. Consistent with proteomic analysis, the metabolic pathway was listed as one of the major pathways involved. Besides, the PI3K-Akt signalling pathway that is involved in the multidrug resistance of cancers was observed.

**TABLE 2:** Top ten upregulated IGPs identified through glycoproteomics analysis.

Upregulated IGPs							
Gene Name	Protein ID	P.Value	Fold.Change	Threshold	N-glycan site	N-glycan	Glycan Site
LG3BP	Q08380	5.18E-09	8.29928459742563	UP	AAIPSALDTJSSK	H(5)N(4)A(1)	551
SAP	P07602	8.51E-10	8.21100856515656	UP	TCDWLPKPJMSASCK	H(5)N(4)	101
CREG1	O75629	2.80E-09	8.07099481273724	UP	VJETEMDIAK	H(5)N(2)	160
P3H1	Q32P28	2.86E-07	8.06769808474273	UP	LLJGSQR	H(5)N(2)	467
SAP	P07602	5.38E-10	8.02656196119227	UP	TCDWLPKPJMSASCK	H(6)N(5)	101
AT1B3	P54709	1.58E-05	7.99999833803474	UP	JLTVCPDGALFEQK	H(7)N(6)A(1)	124
LG3BP	Q08380	9.91E-10	7.96047852749691	UP	AAIPSALDTJSSK	H(7)N(6)	551
LAMP2	P13473	6.34E-07	7.70527367965735	UP	VQPFJVTQGK	H(6)N(3)A(1)	356
ITA3	P26006	7.97E-10	7.60864327489385	UP	TSIPTINMEJK	H(6)N(5)A(1)	969
P3H1	Q32P28	4.13E-10	7.60582638006502	UP	LLJGSQR	H(6)N(2)	467

### 3.4 Conjoint analysis of proteomic and glycoproteomics

**FIGURE 5:**
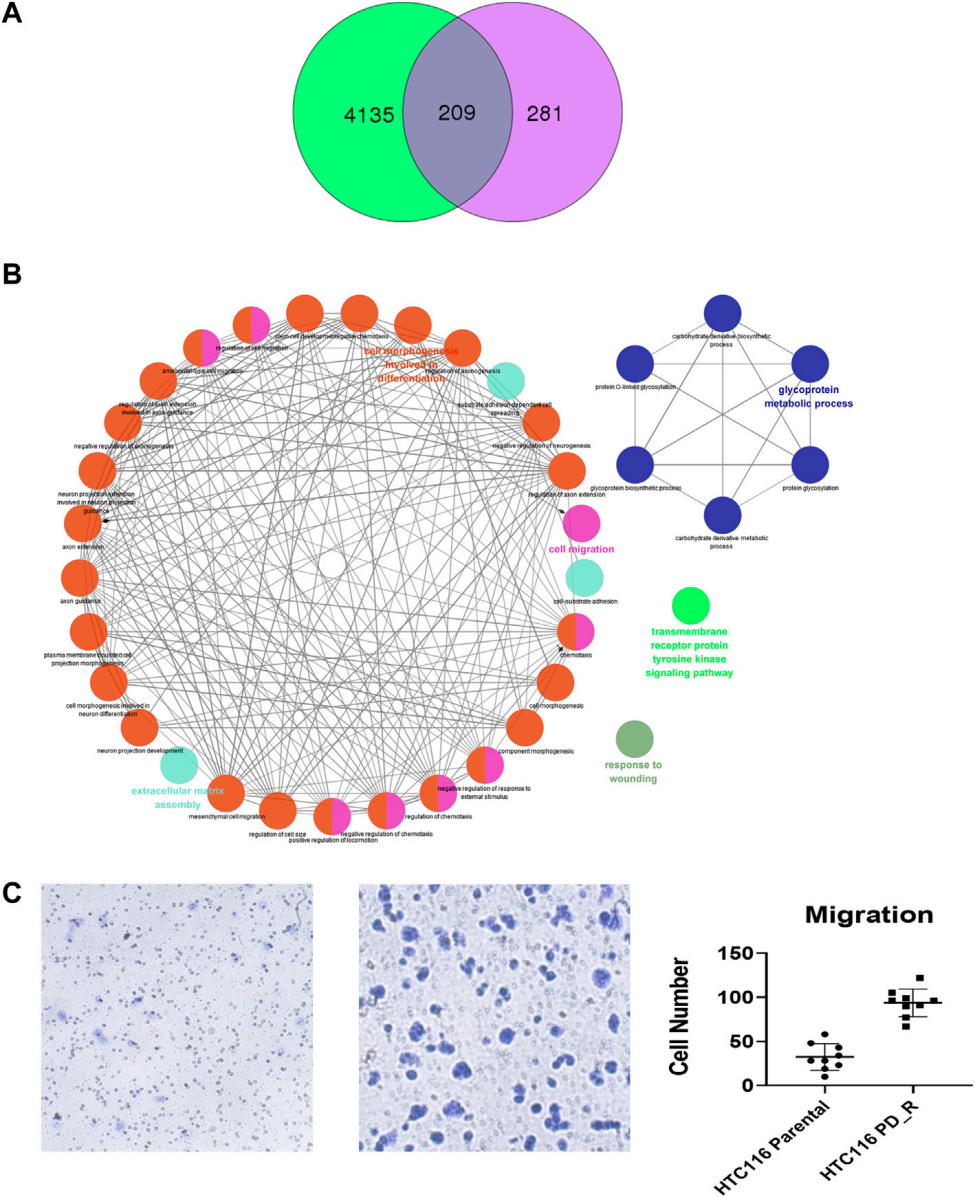
Exclusively identified N-glycoproteins in glycoproteomics analysis. **(A)** Venn diagram of proteins identified in proteomics or glycoproteomics, 281 proteins were exclusively identified in glycoproteomics. Green: Number of proteins identified by proteomics; purple: number of N-glycoprotein identified by glycoproteomics; dark blue: number of proteins identified by both methods. **(B)** The biology process interaction network of 281 N-glycoproteins was constructed by the ClueGO app in Cytoscape software, based on GO data resource. For each module, the most significant pathway is highlighted by a colored name label. The size of the nodes indicates the number of identified proteins within this component. **(C)** Representative images of the transwell migration assay of HCT116 and HCT116 PD_R cells, and the quantitative number of migration cells.

**FIGURE 6:**
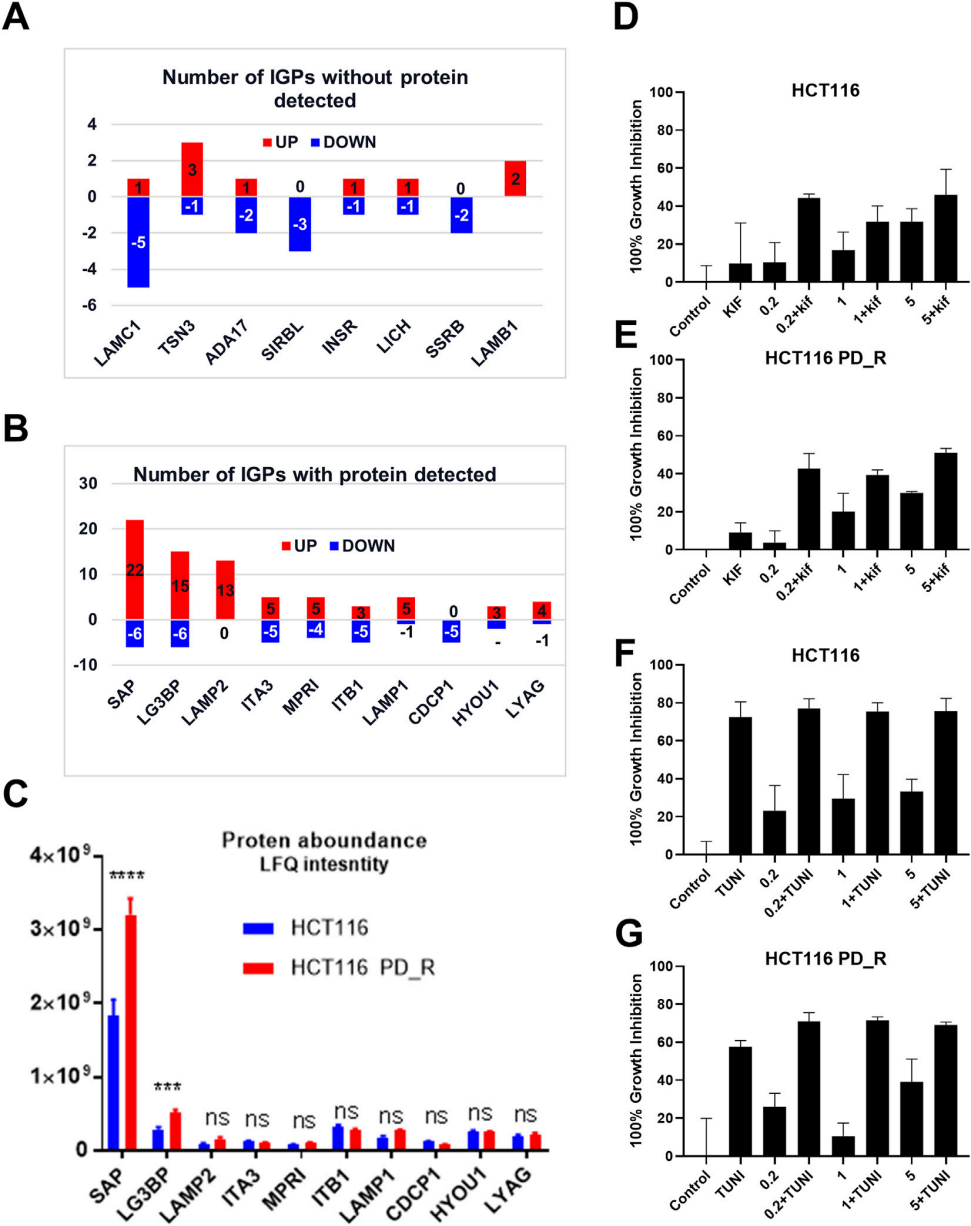
Integration analysis of proteomics and glycoproteomics. **(A)** Number of IGPs for N-glycoproteins exclusively identified in glycoproteomics studies. Red columns represent the numbers of the IGP with upregulation in HCT116 PD_R *versus* HCT116. The Blue column below the *x*-axis represents the downregulated numbers. **(B)** Number of IGPs for N-glycoproteins that were also identified in proteomic analysis. **(C)** The original protein abundance LFQ intensities of corresponding glycoproteins as shown in Figure **(B)**. **(D,E)** Growth inhibition of the combination treatment of kifunensine (30 μM) and palbociclib (0.2, 1, 5 μM) to HCT116, and HCT116 PD_R cells. **(F,G)** Growth inhibition of the combination treatment of tunicamycin (0.1 μM) and palbociclib (0.2, 1, 5 μM) to HCT116, and HCT116 PD_R cells. (Statistical analysis was performed by Graphpad Prism 8.0, and the Two-Way ANOVA multiple comparisons were applied within each row. The significance of the differences was presented by starts: **** means *p*-value <0.0001, *** means *p*-value <0.001, ** means *p*-value <0.01, * means *p*-value <0.05, ns means no significant difference).

As shown in the Venn diagram, 281 proteins were exclusively identified in glycoproteomics analysis (Figure 5A), while only 209 proteins overlap with proteomics analysis. To obtain a general overview of the molecular functions of these N-glycoproteins exclusively identified by glycoproteomics, they were uploaded into the ClueGo app for pathway enrichment analysis and interaction network module analysis. These N-glycosylated proteins were enriched in the pathways of cell migration, extracellular matrix assembly, glycoprotein metabolic process, *etc.*, many of which are known to be associated with the malignancy of tumor cells. We further identified the cell migration capability of resistant cells, with the transwell system we found that the number of migrating cells in resistant cells was much higher than that of the parental cells (Figure 5C). For those N-glycoproteins only identified by glycoproteomics, the N-glycoproteins with more than one IGP identified were listed in Figure 6A; Table 3. The red columns indicated the number of upregulated IGPs of the corresponding N-glycoprotein. LAMC1 had six IGPs, one of them was upregulated in resistant cells with 3.7-fold change, and the other five were downregulated compared to parental cells. For N-glycoproteins detected both by proteomics and glycoproteomics, the up or downregulated IGPs of the top 10 N-glycosylated proteins were presented in Figure 6B. The protein abundances presented as LFQ intensities of these 10 N-glycoproteins were extracted and presented in Figure 6C. Significant differences were observed in SAP and LG3BP according to Student’s t-test analysis, however, the Fold Changes for these two proteins were 0.75 and 0.79 based on Limma-analysis. There was no difference observed for the other eight glycoproteins. It is suspectable that N-glycosylation rather than proteins’ abundance may have a more important role in drug resistance. To verify the role played by glycosylation in palbociclib resistance, glycosylation inhibitors tunicamycin (Tun) and kifunensine (Kif) were used to combine with palbociclib in both parental and resistant cells. Kifunensine of 30 μM and tunicamycin of 0.1 μM were applied to combine with palbociclib (0.2, 1, 5 μM), the results suggested that inhibition of glycosylation with these two drugs can increase the growth inhibition effect of palbociclib both in parental and resistant cells.

**TABLE 3:** DEGs of N-glycoproteins that were exclusively identified in glycoproteomics analysis.

Gene name	Glycan type	Glycan	Glycosite	N-glycopeptide	Fold Change	Expression
LAMC1	High Mannose	H(8)N(2)	1107	VJNTLSSQISR	-7.17402508951035	DOWN
	High Mannose	H(9)N(2)	1107	VJNTLSSQISR	-5.55229753923659	DOWN
	High Mannose	H(8)N(2)	1022	CDQCEENYFYJR	-4.60323061989901	DOWN
	High Mannose	H(8)N(2)	1395	KIPAIJQTITEANEK	-2.33896609247554	DOWN
	High Mannose	H(6)N(2)	1107	VJNTLSSQISR	-1.39544516538119	DOWN
	High Mannose	H(5)N(2)	1107	VJNTLSSQISR	3.70205623797142	UP
TSN3	High Mannose	H(5)N(2)	167	JQSVPLSCCR	-4.68318204639246	DOWN
	High Mannose	H(5)N(3)	167	JQSVPLSCCR	1.74303836743644	UP
	High Mannose	H(3)N(2)	167	JQSVPLSCCR	3.00526887649623	UP
	High Mannose	H(4)N(3)	168	JQSVPLSCCR	4.82106067156722	UP
ADA17	High Mannose	H(5)N(2)	264	JTSWDNAGFK	3.10036857979126	Up
	High Mannose	H(5)N(4)	452	MFSJCSK	-5.51865334828882	Down
	High Mannose	H(7)N(2)	264	JTSWDNAGFK	-5.71158568117761	Down
SIRBL	High Mannose	H(5)N(2)	269; 270	AENQVJVTCQVR	-5.78722918853113	Down
	High Mannose	H(7)N(2)	269; 270	AENQVJVTCQVR	-6.31763596998944	Down
	High Mannose	H(6)N(3)	269; 270	AENQVJVTCQVR	-7.13210424699965	Down
INSR	Sialylated	H(6)N(5)A(1)	445	HJLTITQGK	4.55870285729587	Up
	High Mannose	H(5)N(2)	920	GLSPGJYSVR	-5.35503059802136	Down
LICH	High Mannose	H(7)N(2)	273; 240	NLJMSR	6.96014333143867	Up
	High Mannose	H(3)N(2)	321	NYFHYJQSYPPTYNVK	-6.46998870036861	Down
SSRB	High Mannose	H(9)N(2)	88	IAPASJVSHTVVLRPLK	-9.47950268805305	Down
	High Mannose	H(9)N(2)	88	IAPASJVSHTVVLR	-10.6769372305911	Down
LAMB1	High Mannose	H(6)N(2)	677	GTJYTVR	4.66438842259314	Up
	High Mannose	H(5)N(2)	1279	LSDTTSQSJSTAK	4.1862817227656	Up

## 4 Discussion

Palbociclib is a CDK4/6 inhibitor that was first approved for breast cancer patients (Ettl, 2016; de Dueñas et al., 2018) in 2015, and was also undergoing clinical trials for multiple cancer types including CRC patients (ClinicalTrials.gov: NCT03446157). However, like all other target drugs, palbociclib use may eventually lead to drug resistance (Yang et al., 2017; Aleksakhina et al., 2019; Pandey et al., 2019). Finding out the mechanism behind the development of acquired resistance to target drugs is an urgent need to offer patients better therapy regimes thus prolonging their survival chance. Profound investigation of the potential mechanism underlying drug resistance required new techniques and new designs with obtainable comprehensive high-throughput data. With the recent advance in label-free LC-MS omics analysis, it is expected to identify the new mechanisms for drug resistance with high accuracy and efficiency (Higgs et al., 2008; Sandin et al., 2015). Unlike previous findings, we did not observe significant differences in cyclin proteins including cyclin E in our proteomic data. Firstly, the expression of genes and mRNAs may not transcript to functional proteins after the complex transcription and translation processes. Secondly, the expression level of cyclin E may have a difference in HCT116 and resistant cells, but the expression level of cyclin E might be comparatively low, and the expression of cyclin E had been overwhelmed by other high-abundance proteins. Thirdly, cancers are different, cell lines are different, and the mechanisms developed to be resistant could be different. There may be no difference in cyclin E between resistant and parental HCT116 cells.

Through proteomic analysis, COX-1 in our study was firstly found to be associated with palbociclib resistance (Figure 2A; Table 1). COX-1 has long been reported to be associated with tumorigenesis in multiple cancer types (Rouzer and Marnett, 2009; Pannunzio and Coluccia, 2018). With controversy, the decreased expressed mRNA of COX-1 has been identified in 51 colorectal cancer patients by comparing their tumor tissues and adjacent tissues (Church et al., 2004). However, using COX-1 knockout mice revealed that COX-1 was required from the early stage of intestinal polyp development, which is the first step of developing colorectal cancer. In our study, we found 5 times upregulated expression of COX-1 protein in the palbociclib-resistant CRC cells. As shown in Supplementary Figure S3, the IC50 of resistant cells to COX-1 specific inhibitor SC-560 was significantly decreased compared to the parental cells, namely the resistant cells were a lot more sensitive to COX-1 inhibitor. It was strong evidence suggesting that the proliferation of resistant cells relied more on the COX-1 expression than the parental cells. With this COX-1 inhibitor SC-560, the resistant cells were partially re-sensitized to palbociclib treatment (Figure 2E). This confirms the result of proteomic analysis. Except for COX-1, the second upregulated DEP was NDUFB8, which together with COX-1 involved in the pathway of oxidative phosphorylation (OXPHOS). OXPHOS provides most of the ATP that animals and plants use to support life and maintain metabolic homeostasis (Wilson, 2017). In conventional understanding, cancer cells were represented by up-regulation of glycolysis and downregulated in OXPHOS, since one of the metabolic features of cancer cells is to avidly take up glucose for aerobic glycolysis(Zheng, 2012). Warburg originally proposed that aerobic glycolysis in cancer cells was due to a permanent impairment of mitochondrial OXPHOS. However, with emerging evidence, certain tumor types would develop different metabolism pathways which induced upregulated OXPHOS (Ashton et al., 2018). In this study, upregulated OXPHOS (Supplementary Figure S3) could be developed by HCT116 PD-R cells to defend the impairments from long time palbociclib exposure. As for the KEGG enrichment result, around 100 DEPs were identified to involve in the metabolism pathway (Figure 2C). Further evidence is the GO circle plot (Figure 2D), the metabolism of carbohydrates, lipid, cellular protein, glutathione, and glucose was all upregulated in resistant cells than that in parental cells. With direct mass spectrometry detection under ESI-, we found that ATP and L-glutathione were increased in resistant cells **(** Supplementary Figure S4 **)**. Higher OXPHOS and increased glucose metabolism both suggested upregulation of ATP production. And as the result of GO enrichment of upregulated glutathione metabolism pathway, we verified that the L-glutathione did increase in resistant cells. Glutathione has long been recognized as a feature of drug resistance, it could be conjugated with anti-cancer drugs to form a less toxic and more water-soluble form to export out of the cancer cell (Cazenave et al., 1989; Zhang et al., 1998).

To verify the observation from proteomic results that palbociclib-resistant cells acquired a generally higher metabolism state, we conducted LC-MS metabolomics to investigate the changes in metabolites. Three biology repeats have been conducted, and two study groups could be entirely separated according to the metabolic types and their abundance (Figure 3A). Through data analysis, the number of upregulated features was a lot more than the downregulated ones (Figure 3B), which was consistent with the proteomic finding, that the resistant cells have a general upregulated metabolism status. Within these features, the annotated metabolites were found to be involved in N-glycan biosynthesis and asparagine N-linked glycosylation through KEGG pathway enrichment. Since N-glycosylation of proteins is well known to occur at the asparagine residues(Lowenthal et al., 2016), we hypothesize that the N-glycosylation in palbociclib-resistant cells would be increased.

In the third part of this study, LC-MS/MS glycoproteomics analysis was conducted. Supporting the previous finding, we observed a global elevation of N-glycosylation in resistant cells in terms of the number of intact N-glycopeptides, the number of N-glycoproteins, and the diversity of N-glycosites (Figure 4A). Specifically, the elevated composition of sialylated glycan in resistant cells suggests the malignancy of these cells. Notably, changes in sialylation have been observed following the induction of EMT, a process that allows cancer cells to break away from tumors, migrate and invade (Pinho and Reis, 2015; Bhide and Colley, 2017). The differently expression glycopeptides (DEGs) identified were found to be involved in the lysosome, metabolic pathway as well as PI3K-Akt signaling pathways. As expected, the KEGG term metabolic pathway was observed in this case, this was consistent with the findings from proteomic and metabolomics analysis. Surprisingly, the abnormally expressed PI3K-Akt signaling pathway was here identified by glycoproteomics, which was not observed in previous proteomics analysis. 18 N-glycoproteins that were involved in the PI3K-Akt pathway were identified in the resistant cells. As a well-studied chemoresistance feature(Liu R. et al., 2020), PI3K-Akt was found to be associated with palbociclib-resistant cancer cells as well (Liu et al., 2022). Since the corresponding proteins were not detected, we hypothesize that N-glycosylation may play a more important role in PI3K-Akt-induced drug resistance. To figure out this issue, we integrated and analyzed the proteomic data and glycoproteomic data. To our surprise, almost 1/2 of glycoproteins identified through glycoproteomics were not identified by proteomics. In proteomics analysis, it is common that high-abundance proteins overwhelm those low-abundance ones (Millioni et al., 2011). Through HILIC enrichment, certain low-abundance proteins with important biological functions would be presented. For those 281 N-glycoproteins with low abundances that were not detected in proteomics analysis, we plotted the GO biology process network and found that the cell migration, the glycoprotein metabolic process, the transmembrane receptor protein tyrosine kinase signaling pathway, and the extracellular matrix assembly were enriched. Both cell migration and extracellular matrix assembly suggested an increasing capability of migration was acquired by palbociclib-resistant cells. A transwell assay was performed to verify this result, and a significant increase in the number of migration cells was observed in HCT116 PD_R (Figure 5C; Supplementary Figure S5). The integration analysis could provide a more detailed and comprehensive map to illustrate the mechanisms behind drug resistance.

We further analyzed DEGs with or without protein detected separately. Figure 6A present the most N-glycosylated proteins with their corresponding IGPs. LAMC1 (P11047, Laminin subunit gamma-1) with altered glycosylation pattern was exclusively found by glycoproteomics, it is a kind of protein that participates in signaling pathways involving information of the adherent junction and the basement membrane, and the high expression of LAMC1 is associated with resistance to multiple cancer drugs (Bai et al., 2022). However, the role played by glycosylation LAMC1 was not mentioned in previous studies. ADA17 (ADAM17) at the protein level was reported to induce drug resistance through hypoxia through PI3K/Akt pathway (Wang et al., 2013; Zhang et al., 2018). For SIRBL (SIRPB1), there was no direct study reported that it played a role in drug resistance, it was indeed found to promote cell proliferation *via* Akt activation(Song et al., 2020), which was evidence of drug resistance, since cancer cells normally become more malignant with the higher capability of proliferation, migration, *etc.* Upon the development of drug resistance.

Next, DEGs with detectable proteins were compared in Figures 6B, C, the number of IGPs were generally more than those without protein detection. The most glycosylated N-glycoprotein was SAP (Figure 6B; Supplementary Table S10), it had 22 upregulated and six downregulated IGPs. SAP (P07602, prosaposin) is a mitochondria protein that plays a critical role in sphingolipid and cancer metabolism (Zhang. J et al., 2020). Researchers used the TMT label method to identify glycoproteins from cancer cells and found that prosaposin could suppress immune cell infiltration, resulting in the promotion of cancer (Miyahara et al., 2022). In our study, the protein abundance of prosaposin has a significant difference between the 2 cell groups (*p*-value < 0.001), but the Fold Change was only 0.75 defined by limma analysis (Supplementary Table S4), which was not as dramatic as the those have fold-changes above four or 5. In this case, we suspected that protein abundance alteration may less important than the glycosylation changes of prosaposin in the development of palbociclib resistance. For LG3BP (Q08380), resistant cells had 15 upregulated and six downregulated IGPs, the number of IGPs was second to SAP. However, the fold-change of a sialylated type (H(5)N(4)A(1)) glycosylation at site AAIPSALDTJSSK of LG3BP was 8.299, which is the most upregulated IGP with the highest Fold Change (Table 2). LG3BP (Galectin-3-binding protein) can promote integrin-mediated cell adhesion and it plays an important role in mediated tumor cell survival during the metastatic process (Iacobelli et al., 1994; Lin et al., 2015). LG3BP is a highly glycosylated protein and was expected to be the target of glycosylation-mediated cancer drug delivery (Cai et al., 2018). Similar to SAP, the protein expression of LG3BP was slightly upregulated in resistant cells with a not obvious Fold Change. For the rest N-glycoproteins in Figure 6C, there was no statistical difference in the protein abundance between resistant and parental cells. LAMP1 (Lysosome-associated membrane protein-1) is a surface cell protein that attacks cancer researchers' interests for a long time, which is involved in migration and invasion functions in metastatic tumor cells at protein level (McCormick et al., 1998; Betts et al., 2003; Alter et al., 2004). However, investigations focused on the function of the glycosylation of LAMP1 were rarely observed. An animal study with mice found that abnormal LAMP1 glycosylation was potentially associated with Niemann-Pick disease (Cawley et al., 2020), but none of this kind of studies were found in cancer research. Generally speaking, the highly altered glycosylation of these 10 proteins (Figures 6A, B) in terms of the diversity and the abundance of IGPs suggested that glycosylation might play a more important role than protein abundance in the development of drug resistance.

The important role played by glycosylation of proteins in the process of cancer development was widely accepted and researchers started to suggest glycosylation of proteins could be an effective strategy to improve cancer therapy (Moradi et al., 2016; Cai et al., 2018). Glycosylation is also associated with multi-drug resistance in several kinds of cancer types (Li et al., 2013; Wu et al., 2016; Wu et al., 2018; Zeng et al., 2021). In the last part, we have included two glycosylation inhibitors tunicamycin and kifunensine to combine with palbociclib, and found that with the suppression of glycosylation, the palbociclib resistance could be partially reversed. This finding further supported our hypothesis based on glycoproteomics that elevated glycosylation was associated with the development of palbociclib resistance. Tunicamycin is a potent glycosylation inhibitor that inhibits the very first step of entire glycan synthesis (Wu et al., 2018). Wus' work found that inhibition of glycan synthesis by tunicamycin could induce cell death in multi-drug resistant cancer cells. On the other hand for kifunensine, it is a selective inhibitor for α-mannosidase I, it could result in the accumulation of Man7-9GlcNAc2 high-mannose type oligosaccharides on glycoproteins (Zhou et al., 2008). Comparing to tunicamycin, the synergistic effect of kifunensine was more dramatic as in Figures 6D, E. This suggested that high mannose glycosylation might play a major role in the development of palbociclib resistance in HCT116 cells.

## 5 Conclusion

In this work, we have hired label-free LC-MS based omics studies to investigate the unknown mechanism behind palbociclib resistance in HCT116 cells. Through proteomic analysis, we found the metabolism-related pathways were generally upregulated in palbociclib resistance cells. And then metabolomics study was performed to reveal the increase of N-glycan biosynthesis-related in resistant cells. Afterward, a global N-glycosylation in the resistant cells was indeed identified to be elevated through glycoproteomics. At the final step, glycosylation inhibitors were hired to conclude that glycosylation inhibition could reverse the acquired palbociclib resistance. This study was carried out logically, following the result initially identified through proteomics analysis one step by one step. The integration analysis with proteomic and glycoproteomic, and the application of glycosylation inhibitors together suggested that inhibition of glycosylation could be an effective approach to reverse acquired drug resistance. It is expected that the label-free LC-MS technique could be widely used to investigate cancer drug resistance to offer a new solution to improve therapeutic effects for patients.

## 6 Limitations

Firstly, the annotation of metabolites remained to be a huge challenge in metabolomics analysis. Efforts have been made continuously, however, due to the complex composition of different chemical structures, it is still hard to automatically annotate all candidate features correctly. In this study, among all the different expressed features, only a limited number of metabolites were identified. However, these results were successfully verified by following a glycoproteomic study afterward. It should be long-term work to establish approaches to improve the result of metabolomics studies in the future, further effort should be put into the optimization of pretreatment methods, updating the equipment used, and building up new bioinformatic strategies.

Secondly, the functions of N-glycoproteins were rarely studied in previous reports. Currently, researchers have been dedicated to investigating the biological roles represented by the expression of proteins rather than their glycosylation situation. It is a comparatively new area with technique challenges. Here we used glycosylation inhibitors to suppress the overall glycosylation of protein and proved that N-glycosylation was associated with drug resistance. However, studies that focus on the N-glycosylation of specific proteins were encouraged.

## Data Availability

The mass spectrometry proteomics data have been deposited to the ProteomeXchange Consortium (http://proteomecentral.proteomexchange.org) *via* the iProX partner repository [76] with the dataset identifier PXD038509.
