# Supplementary material for: Investigation on acquired palbociclib resistance by LC-MS based multi-omics analysis
Source: Front Mol Biosci. 2023 Jan 19;10:1116398. doi: 10.3389/fmolb.2023.1116398 (PMC9892630; doi:10.3389/fmolb.2023.1116398)
Supplement: Supplementary file 1 [file DataSheet1.zip › Supplementary Figures.docx]

Supplementary Figure S 1


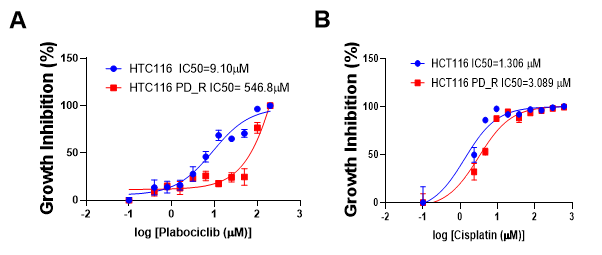


Supplementary Figure 1: Growth inhibition of HCT116 and HCT116 to palbociclib and Cisplatin, the IC50 was calculated with GraphPad Prism 7.04 with function of log(inhibitor) vs. response (three parameters)

Supplementary Figure S 2


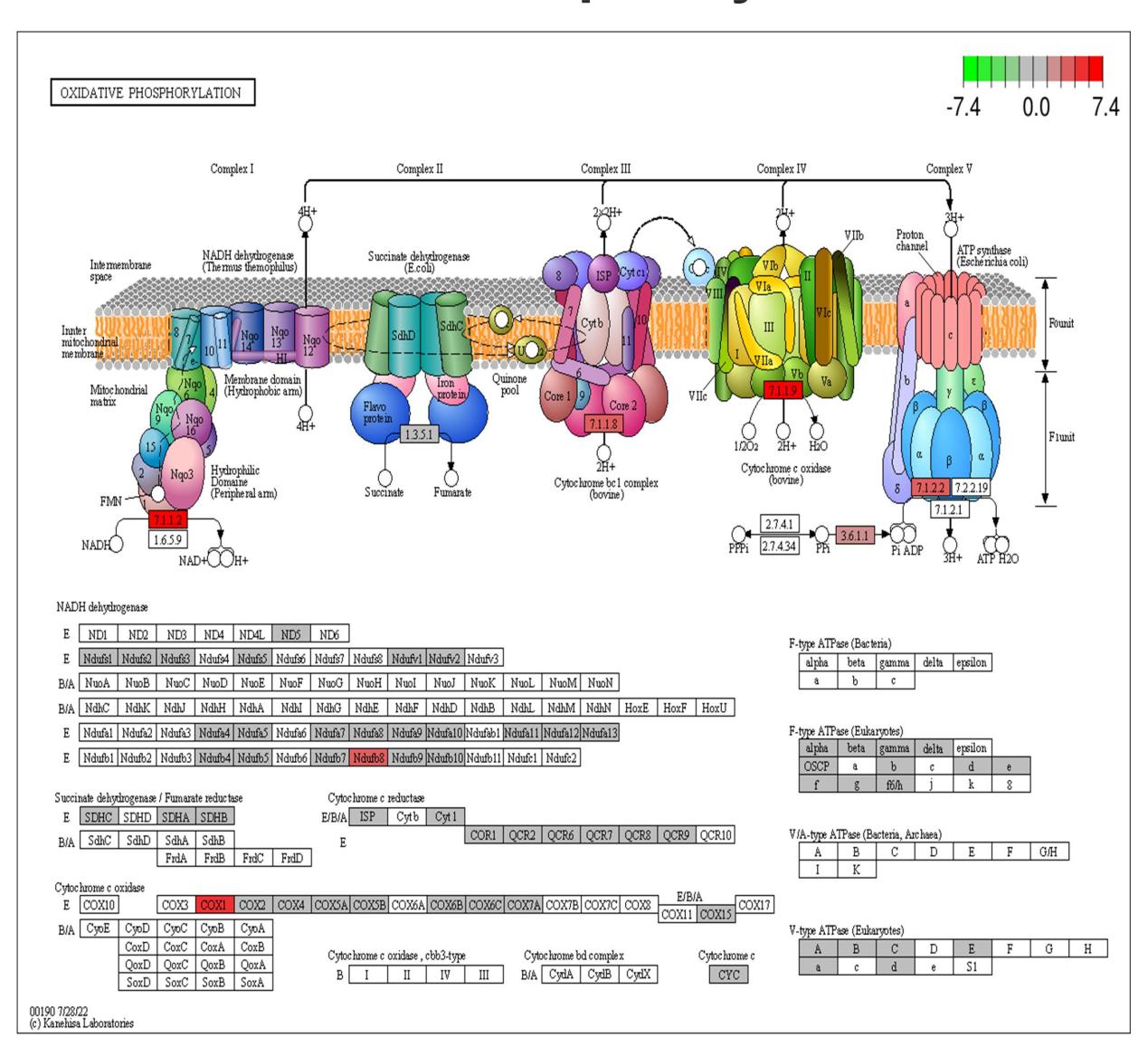


Supplementary Figure 2: KEGG pathway of oxidative phosphorylation. Red nodes: proteins upregulated in HCT116 PD_R compared to HCT116; Grey nodes: the proteins without significant change in expressing level, or not identified in this study; Green nodes: proteins downregulated.

Supplementary Figure S 3

Supplementary Figure 3: Growth inhibition SC-560 for HCT116 and HCT116 PD_R cells.

Supplementary Figure S 4


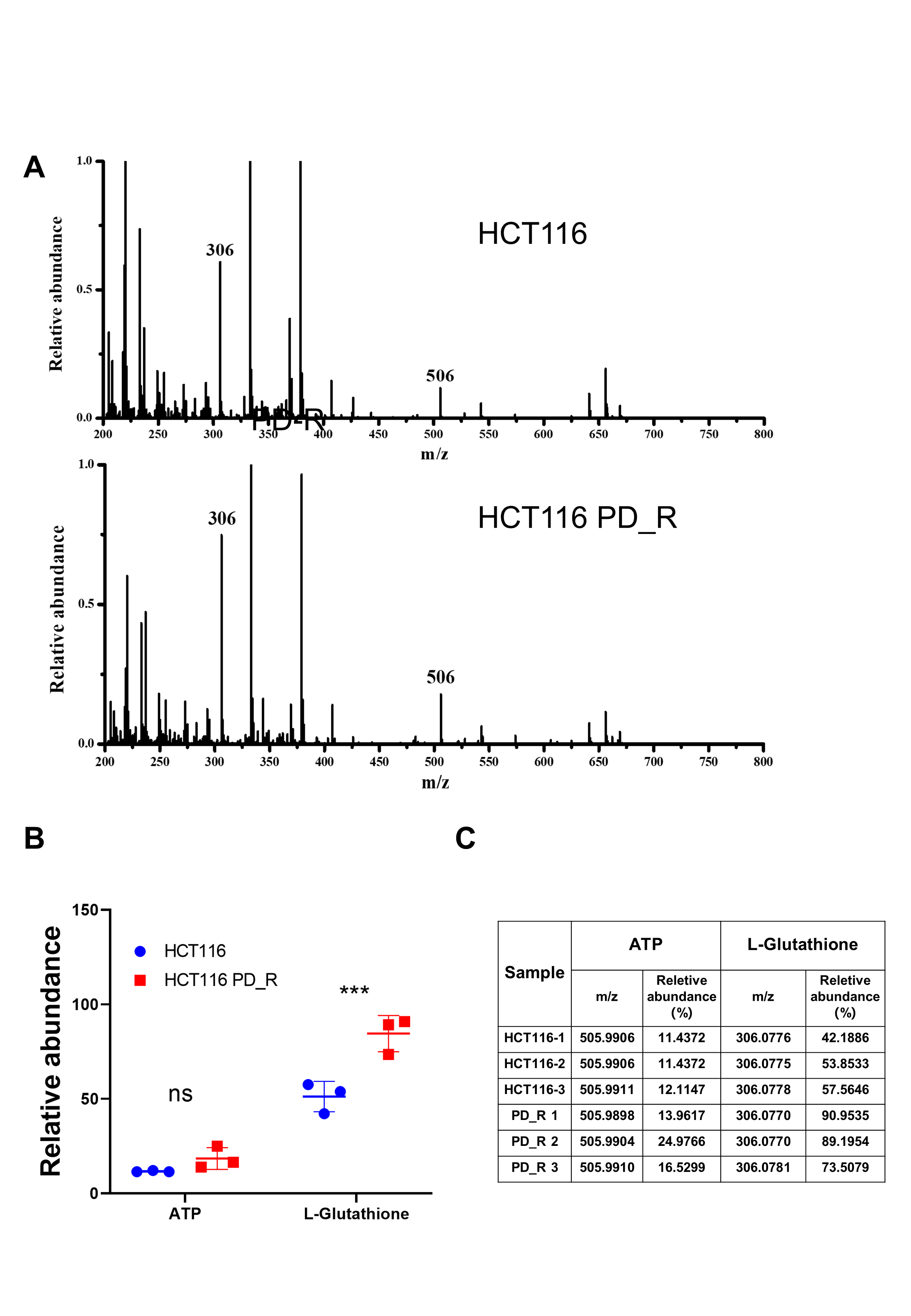


Supplementary Figure 4: Relative abundance of ATP and Glutathione. (A) MS figure of HCT116 and HCT116 PD_R under detection of ESI-. (B) Dot plot of ATP and Glutathione. (C) Original data extract for raw files.

Supplementary Figure S5


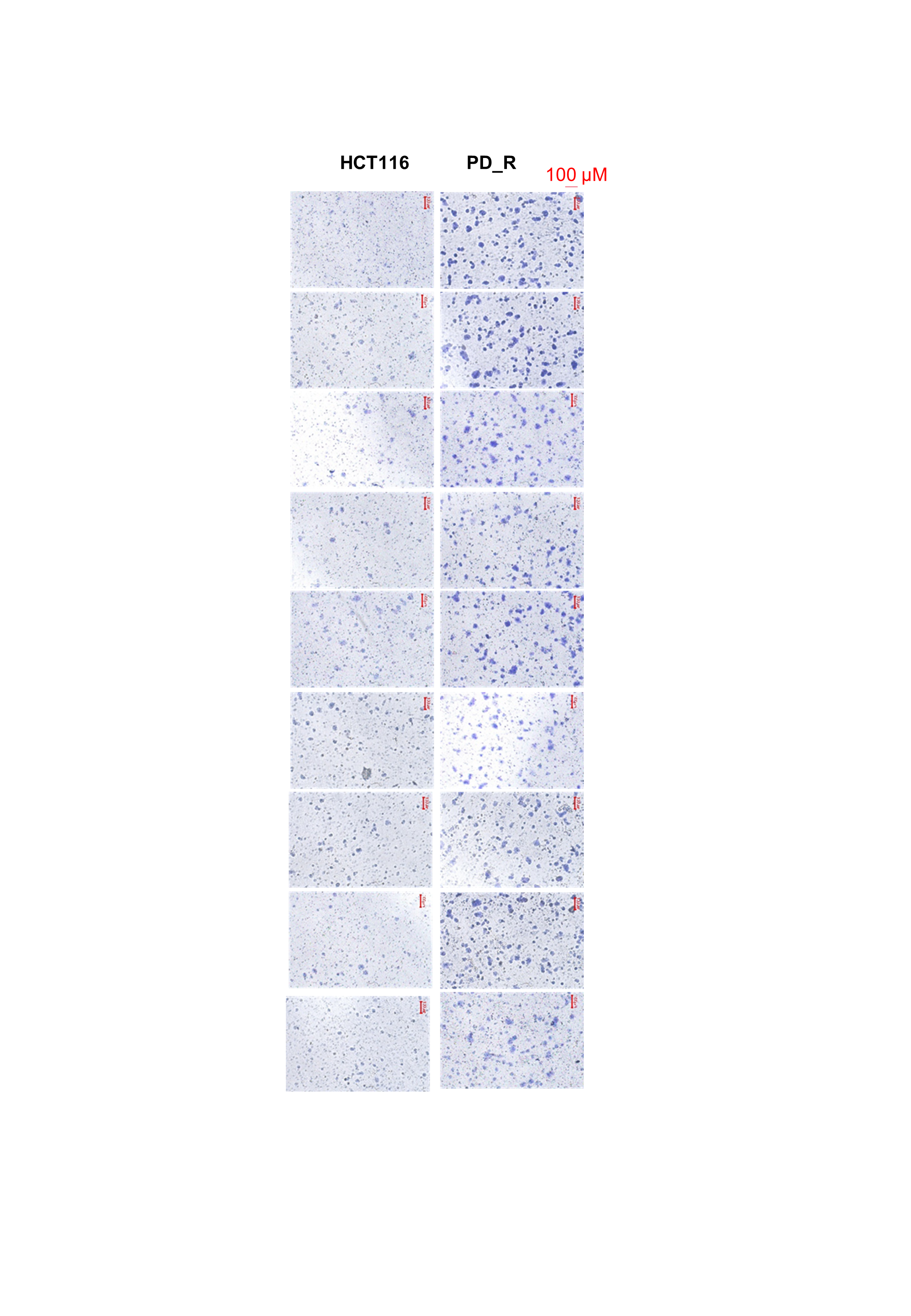


Supplementary Figure 5: Figures of transwell migration assay. Three biology repeats were set for the transwell study, and 3 random views were selected for each repeat.
